# New Insight in the Assessment of Atrial Size and Function

**DOI:** 10.1155/2015/310846

**Published:** 2015-11-08

**Authors:** Giovanni Di Salvo

**Affiliations:** King Faisal Specialist Hospital & Research Center, Heart Center, Alfaisal University, MBC-16, P.O. Box 3354, Riyadh 11211, Saudi Arabia

The atrium has been considered for too many years a neglected chamber and because of this the assessment of atrial function is still a challenge.

More recently, new noninvasive diagnostic modalities have shed new light on atrial role in several diseases.

The assessment of atrial function may have particular relevance for the growing epidemic of atrial fibrillation which currently affects nearly 2% of the general population, with prevalence expected to double by 2050.

A better knowledge and understanding of atrial function in different cardiac diseases (mitral valve disease, cardiomyopathies, coronary artery disease, heart failure, hypertension, congenital heart diseases, etc.) may have a significant clinical impact in the management and in the prognostic evaluation of our patients.

This special issue focuses on original research articles and review papers that address a broad range of mechanisms associated with atrial dysfunction, with possible prognostic and therapeutic interventions.

In this dedicated issue, R. Leischik et al. reviewed in detail the importance and pitfalls of advanced echo modalities such as strain technology and 3D echocardiography for the analysis of left atrial mechanics.

Strain imaging [1] seems to be the most promising technology for the direct evaluation of left atrial function [2]. However, the lack of dedicated software, standards for acquisition, and analysis limit the use of this technique.

P. Kuchynka et al. reviewed the role of cardiac magnetic resonance and computed tomography in assessment of left atrial size and function, especially before and after atrial fibrillation ablation.

Cardiac magnetic resonance is considered to be the gold standard for volumetric assessment of left atrial size. However, the cost and the availability of the technique do not seem to justify the potential added role when compared to 2D and 3D echo. Late gadolinium enhancement has the ability to tissue-characterize the atria and this may be used for optimizing the treatment of patients suffering from atrial fibrillation. However, the very promising results obtained by strain imaging in detecting atrial fibrosis may represent a valid alternative. Cardiac CT is considered the method of choice for evaluation of pulmonary vein and left atrial anatomy before catheter ablation procedures for atrial fibrillation and for detection of pulmonary vein stenosis after ablation. However, the exposition to ionizing radiations should be carefully taken into account.

D. Regazzoli et al. presented a comprehensive analysis of the potential role of left atrial appendage occlusion in stroke prevention. The left atrial appendage is considered the “most lethal human appendage” as it may cause significant mortality and morbidity in AF patients. In this regard, left atrial appendage occlusion could be an interesting and effective procedure in thromboembolism prevention in atrial fibrillation especially in certain categories of patients.

It is of note that G. de Maat et al. in the present issue demonstrated the effect of left atrial appendage amputation on atrial mechanics.

Finally, C. Santosa et al. presented data on the potential utility of combined 3D left atrial volume measurement and peak E wave mitral flow velocity as echocardiographic guides for acute volume resuscitation.

Taken together, the papers in this special issue present the recent developments and future perspectives in the morphological and functional evaluation of the atria and new possible therapeutic approaches. Importantly, these papers also unmask many challenging issues that should be overcome to improve the evaluation of atrial function as well as the new proposed therapeutical approaches.

*Giovanni Di Salvo*
